# Supplementary material for: SARS-CoV-2 Seropositivity in Nursing Home Staff and Residents during the First SARS-CoV-2 Wave in Flanders, Belgium
Source: Viruses. 2024 Sep 14;16(9):1461. doi: 10.3390/v16091461 (PMC11437469; doi:10.3390/v16091461)
Supplement: Supplementary file 1 [file viruses-16-01461-s001.zip › viruses-3094054-supplementary.pdf]

Supplement S1. Seroprevalence and cumulative RT-PCR positivity per nursing home.

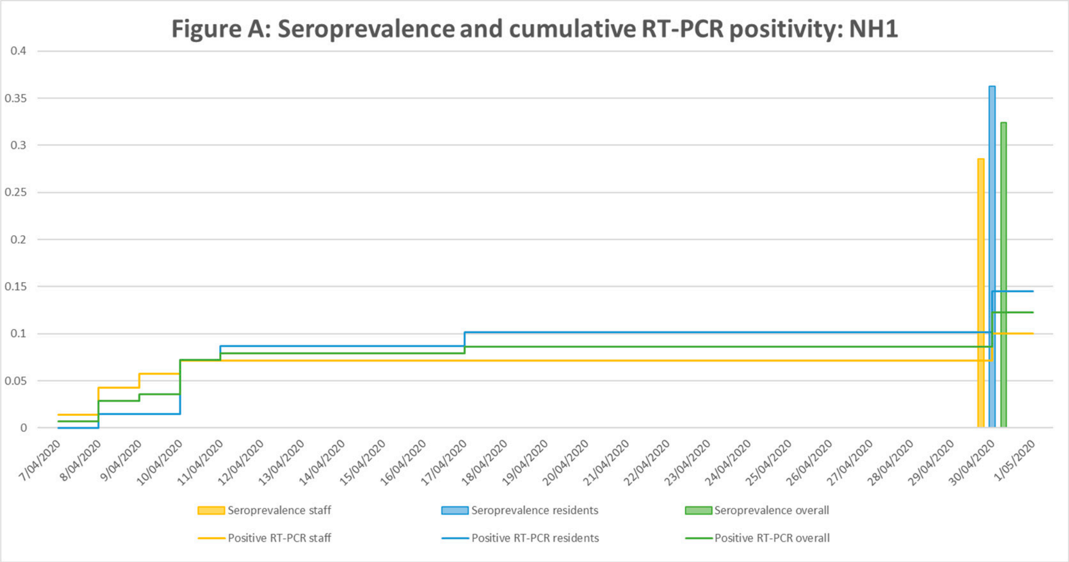

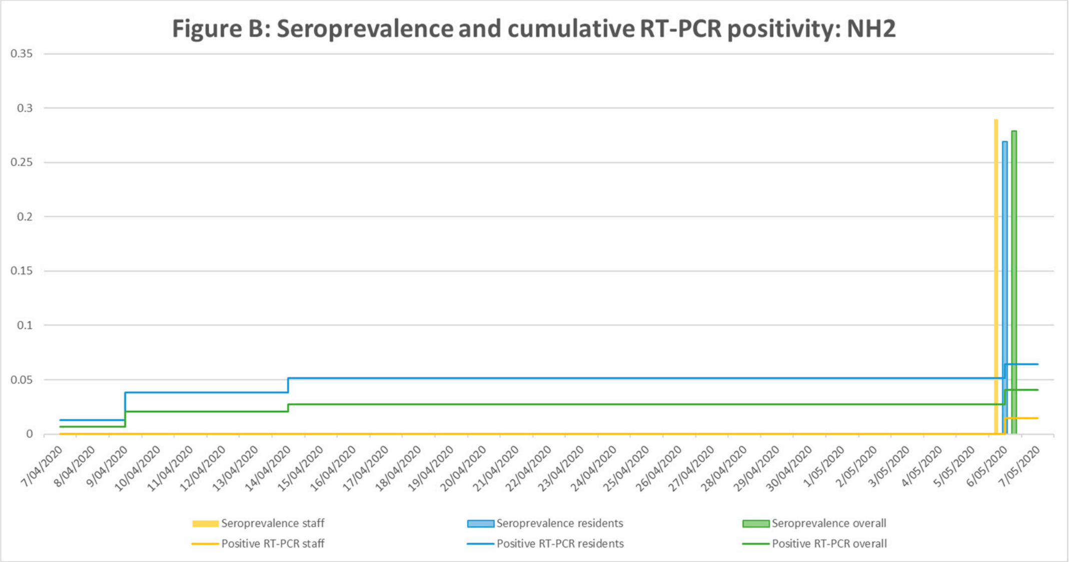

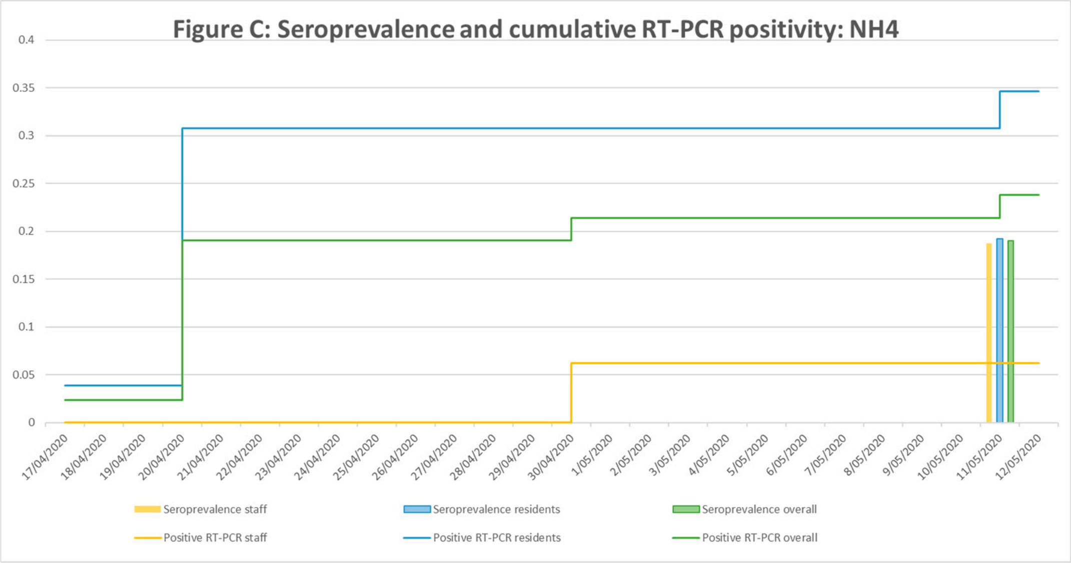

Figure D: Seroprevalence and cumulative RT-PCR positivity: NH7

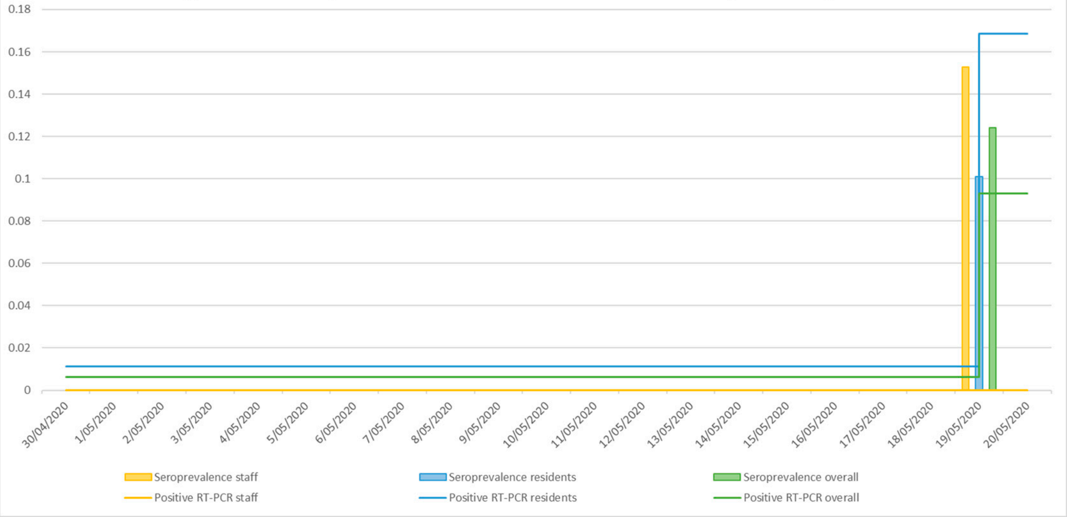

Figure E: Seroprevalence and cumulative RT-PCR positivity: NH8

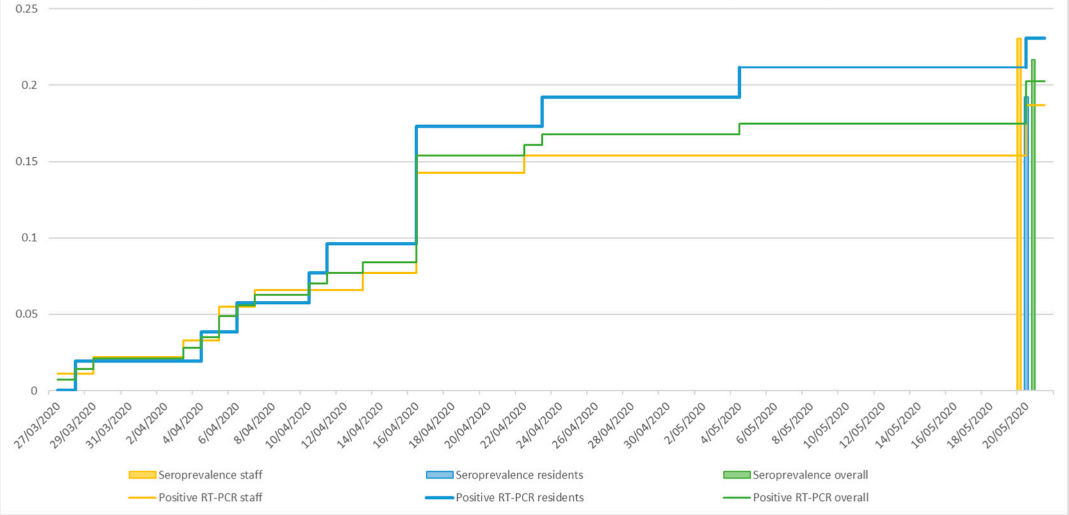

The seroprevalence was compared to the cumulative RT-PCR positivity in a sub-cohort of five nursing homes. The cumulative RT-PCR positivity was calculated over time as the total sum of all positive RT-PCR-test results proportional to the size of the nursing home. The seroprevalence was calculated as the frequency of positive antibody tests proportional to the sample size of the nursing home.

NH, nursing home.

Supplement S2. Cumulative RT-PCR positivity and seroprevalence per nursing home (NH) by date.

Nursing home	Date	Cumulative RT-PCR positivity			Seroprevalence		
		Residents, n (%)	Staff, n (%)	Overall, n(%)	Residents, n (%)	Staff, n (%)	Overall, n(%)
**NH1**	7/04/2020	1/70 (1%)	0/69 (0%)	1/139 (1%)			
	8/04/2020	3/70 (4%)	1/69 (1%)	4/139 (3%)			
	9/04/2020	4/70 (6%)	1/69 (1%)	5/139 (4%)			
	10/04/2020	5/70 (7%)	5/69 (7%)	10/139 (7%)			
	11/04/2020	5/70 (7%)	6/69 (9%)	11/139 (8%)			
	17/04/2020	5/70 (7%)	7/69 (10%)	12/139 (9%)			
	30/04/2020	7/70 (10%)	10/69 (14%)	17/139 (12%)	20/70 (29%)	25/69 (36%)	45/139 (32%)
**NH2**	7/04/2020	0/69 (0%)	1/78 (1%)	1/147 (1%)			
	9/04/2020	0/69 (0%)	3/78 (4%)	3/147 (2%)			
	14/04/2020	0/69 (0%)	4/78 (5%)	4/147 (3%)			
	6/05/2020	1/69 (1%)	5/78 (6%)	6/147 (4%)	20/69 (29%)	21/79 (27%)	51/148 (28%)
**NH4**	17/04/2020	0/16 (0%)	1/26 (4%)	1/42 (2%)			
	20/04/2020	0/16 (0%)	8/26 (31%)	8/42 (19%)			
	30/04/2020	1/16 (6%)	8/26 (31%)	9/42 (21%)			
	11/05/2020	1/16 (6%)	9/26 (35%)	10/42 (24%)	3/16 (19%)	5/26 (19%)	8/42 (19%)
**NH7**	30/04/2020	0/72 (0%)	1/89 (1%)	1/161 (1%)			
	19/05/2020	0/72 (0%)	15/89 (17%)	15/161 (9%)	11/72 (15%)	9/89 (10%)	20/161 (12%)
**NH8**	27/03/2020	1/91 (1%)	0/52 (0%)	1/143 (1%)			
	28/03/2020	1/91 (1%)	1/52 (2%)	2/143 (1%)			
	29/03/2020	2/91 (2%)	1/52 (2%)	3/143 (2%)			
	3/04/2020	3/91 (3%)	1/52 (2%)	4/143 (3%)			
	4/04/2020	3/91 (3%)	2/52 (4%)	5/143 (3%)			
	5/04/2020	5/91 (5%)	2/52 (4%)	7/143 (5%)			
	6/04/2020	5/91 (5%)	3/52 (6%)	8/143 (6%)			
	7/04/2020	6/91 (7%)	3/52 (6%)	9/143 (6%)			
	10/04/2020	6/91 (7%)	4/52 (8%)	10/143 (7%)			
	11/04/2020	6/91 (7%)	5/52 (10%)	11/143 (8%)			
	13/04/2020	7/91 (8%)	5/52 (10%)	12/143 (8%)			
	16/04/2020	13/91 (14%)	9/52 (17%)	22/143 (15%)			
	22/04/2020	14/91 (15%)	9/52 (17%)	23/143 (16%)			
	23/04/2020	14/91 (15%)	10/52 (19%)	24/143 (17%)			
	4/05/2020	14/91 (15%)	11/52 (21%)	25/143 (17%)			
	20/05/2020	17/91 (19%)	12/52 (23%)	29/143 (20%)	21/91 (23%)	10/52 (19%)	31/143 (22%)

Supplement S3. Questionnaire to assess the risk of SARS-CoV-2 self-reported (re)infections in seropositive and -negative participants.

STUDY CODE participant: _ _ _ _ _ _

All questions are applicable to the period between data sampling of seroprevalence on XX/XX/2020 and before first dose of vaccination.

1. Have you experienced one or more COVID-19 infections during this period? (Circle your answer)
    - i. YES - NO
    - ii. DATE(S): ……………………………
   - b. Did the infection meet the case definition of Sciensano?
     - i. YES – NO – I DON'T KNOW
     - ii. https://covid-19.sciensano.be/nl/covid-19-gevalsdefinitie-en-testing
   - c. Did you have one or more positive PCR test(s) (+ date)?
     - i. YES + DATE(S): ………………..
     - ii. NO
   - d. Did you have one or more positive antigen rapid test(s) (+ date)?
     - i. YES + DATE(S):..............
     - ii. NO
   - e. Was the diagnosis radiologically confirmed?
     - i. YES + DATE(S):..............
     - ii. NO
2. In case of a COVID-19 infection, did you require oxygen? (Circle your answer)
    - i. YES – NO
3. Were you hospitalized during this period? (Circle your answer)
   - a. YES + DATE: ………………..
     - i. Was this due to a COVID infection or for another reason?……………………………………………..……………………… ………………………
   - b. NO
4. Are you known to have one or more of the following conditions? (Check all that apply)
   - o Cardiovascular diseases
   - o Diabetes
   - o High blood pressure (hypertension)
   - o Severe lung/heart/kidney disease
   - o Weakened immune system (immunosuppressive conditions) or weakened immune system due to prolonged use of immunosuppressive medication (e.g., chemotherapy, transplant patients, immunosuppressive rheumatoid medication, corticosteroids, etc.)
   - o Active cancer condition
   - o I am not known to have a chronic condition

- Other:
  …………………………………………………………………………………………
  ………..

5. For the nursing home staff: have you worked in a COVID-19 cohort ward in your nursing home? (Circle your answer)
   a. YES - Date(s): ....
   b. NO
6. Was there a time when you did not have sufficient personal protective equipment available? (Circle your answer): YES – NO
